# Technical Considerations for the Generation of Adoptively Transferred T Cells in Cancer Immunotherapy

**DOI:** 10.3390/cancers8090086

**Published:** 2016-09-20

**Authors:** Anthony Visioni, Joseph Skitzki

**Affiliations:** 1 Department of Surgical Oncology, Roswell Park Cancer Institute, Buffalo, NY 14263, USA; anthony.visioni@roswellpark.org; 2 Department of Immunology, Roswell Park Cancer Institute, Buffalo, NY 14263, USA

**Keywords:** immunotherapy, cellular immunotherapy, adoptive transfer, T cell therapy, tumor-infiltrating lymphocyte, tumor-draining lymph node

## Abstract

A significant function of the immune system is the surveillance and elimination of aberrant cells that give rise to cancer. Even when tumors are well established and metastatic, immune-mediated spontaneous regressions have been documented. While there are have been various forms of immunotherapy, one of the most widely studied for almost 40 years is adoptive cellular immunotherapy, but its success has yet to be fully realized. Adoptive cell transfer (ACT) is a therapeutic modality that has intrigued physicians and researchers for its many theoretical benefits. Preclinical investigations and human trials have utilized natural killer (NK) cells, dendritic cells (DC), macrophages, T-cells or B-cells for ACT with the most intense research focused on T-cell ACT. T-cells are exquisitely specific to the target of its T-cell receptor (TCR), thus potentially reducing the amount of collateral damage and off-target effects from treatment. T-cells also possess a memory subset that may reduce the risk of recurrence of a cancer after the successful treatment of the primary disease. There are several options for the source of T-cells used in the generation of cells for ACT. Perhaps the most widely known source is T-cells generated from tumor-infiltrating lymphocytes (TILs). However, studies have also employed peripheral blood mononuclear cells (PBMCs), lymph nodes, and even induced pluripotent stem cells (IPSCs) as a source of T-cells. Several important technical considerations exist regarding benefits and limitations of each source of T-cells. Unique aspects of T-cells factor into their ability to be efficacious in ACT including the total number of cells available for ACT, the anti-tumor efficacy on a per cell basis, the repertoire of TCRs specific to tumor cells, and their ability to traffic to various organs that harbor tumor. Current research is attempting to unlock the full potential of these cells to effectively and safely treat cancer.

## 1. Introduction

The relationship between cancer and the immune system has been recognized as far back as 1909, when Paul Ehrlich proposed that the immune system suppresses tumor formation by a mechanism that would be coined “immune surveillance” [1]. This process has been the subject of research for decades and has been refined into the concept of “cancer immunoediting” [2]. The interplay of the immune system with cancer cells is comprised of interactions in which the immune system functions to protect and propagate cancer cells as well as cause their elimination [3]. As our understanding of this complex relationship has unfolded, immunotherapy of cancer has been an area of parallel research. In general, immunotherapy can be defined as either nonspecific stimulation of the immune system, active immunization, or adoptive cell transfer (ACT) [4]. ACT has been the subject of continued research ever since Rosenberg and colleagues first reported their experience with lymphokine-activated killer cells (LAK) and tumor-infiltrating lymphocytes (TIL) [5]. In particular, T-cell ACT has been the most widely studied.

ACT has sparked interest due to several theoretical and realized benefits. ACT has the potential to be relatively non-toxic, which is due to two main reasons. First, the cells used in ACT are all autologous. Every ACT protocol to date uses the patient’s cells to derive their cancer treatment. Second, immune cells have the power to be exquisitely specific. Indeed, the T-cell receptor (TCR) present on the T-cell surface is specific to its cognate peptide in the context of an MHC molecule, which can limit its toxicity. In general, ACT with T-cells can be divided into 3 stages: obtaining autologous cells, ex vivo manipulations and expansion, and infusion back into the patient. The focus of this review will be the technical aspects of the generation of T-cells and therefore, obtaining the cells and ex vivo manipulations.

## 2. Sources of Autologous Cells

TIL are one of the oldest and best studied forms of T-cell ACT. As the name denotes TIL are the lymphocytes that have trafficked to a tumor and are present within the tumor or at the periphery. These cells are an obvious choice for use in ACT, since their presence in proximity to the tumor suggests a level of reactivity against the tumor. Some lymphocytes have been identified as immunosuppressive and shown to support tumor growth (e.g., T-regulatory cells), but the presence of CD8^+^ T-cells infiltrating the tumor suggests some degree of anti-tumor response [6]. One of the goals of ACT is to remove these cytotoxic T-cells from the immunosuppressive environment of the tumor and re-establish their ability to kill tumor cells.

In order to obtain TIL, a patient must have a tumor that is resectable. This is typically accomplished in the case of melanoma where metastatic disease is often present in the skin or subcutaneous tissues, however, this does pose an obstacle when considering ACT for certain visceral cancers. The group with the most experience in regards to TIL therapy is undoubtedly the Surgery Branch of the National Cancer Institute, where Rosenberg and colleagues have recently published their series of patients with metastatic melanoma who underwent TIL therapy [7]. They report a fairly impressive complete response rate of 40% in patient undergoing the most aggressive lymphodepleting pre-conditioning. These complete responses are also durable with regression still ongoing in 95% of patient at 37 to 82 months. Importantly, 90% of these complete responses were achieved with one treatment of TIL and the remaining 10% of patients only received 2 treatments. This study also examined which factors potentially contribute to a complete response and found three in particular: Increased telomere length, higher percentage of CD8^+^CD27^+^ cells used for therapy, and higher percentage of infused cells present in the peripheral blood at 1 month after therapy. These factors are all associated with persistence of the adoptively transferred cells.

Some groups have attempted to overcome the limitations in obtaining TIL from inaccessible tumors. These researchers have looked at an easily accessible location of T-cells: lymph nodes. A major function of the lymph node is to serve as a site where antigen presenting cells (APC) process and display antigens to a large repertoire of T-cell receptors. This interaction will then coordinate an appropriate immune response in a healthy individual. Unfortunately in cancer, this natural priming of a lymph node is often abrogated by inefficient activation of T-cells by APC that lead to paradoxical tolerance of the cancer cells or by other immunosuppressive factors [8]. Therefore, several studies have examined priming lymph nodes with tumor vaccines and immune stimulating adjuncts to generate lymph nodes enriched for tumor-specific T-cells. Chang and colleagues published their data on vaccine-primed lymph nodes used in ACT for melanoma and renal cell cancer [9]. Although no complete responses were found in melanoma patients, about 17% of RCC patient experienced a complete response to therapy.

One of the issues presented in this study was that the melanoma patients may not have received an adequate number of cells. However, other concerns arise when considering vaccine priming of lymph nodes. As some protocols use irradiated autologous tumor, there is still a requirement for the patient to have a tumor that can be excised. Protocols that do not use autologous tumor typically rely on a single antigen or mixture of a few antigens to prime lymph nodes which may narrow the therapeutic targets that lymphocytes are primed against. Also, most of these protocols inoculate patients a single time prior to removing a lymph node. A single exposure to antigen may not allow enough priming of lymph nodes for full therapeutic efficacy. Finally, by priming a lymph node via vaccination, there may be alterations in signaling to lymphocytes that affect cellular homing to tumor sites [10]. Therefore, antigen-specific cells may be generated but not able to traffic effectively to various tumor sites.

One way to address these concerns is to use lymph nodes that are draining a naturally occurring tumor or tumor draining lymph nodes (TDLN). In theory, TDLN are exposed almost continually to antigen from the tumor in a naturally occurring manner. They are, unfortunately, also under the immunosuppressive effects of the tumor which does not allow for an appropriate immune response [8]. A study by Shu demonstrated progressively growing tumors generated a population of “pre-effector” lymphocytes in TDLN that were functionally impaired, likely due to the immunosuppressive and tolerogenic effects of the tumor [11]. However, several studies have shown that TLDN ACT can be efficacious in mouse models [12,13,14]. Pre-clinical human studies have also shown that TDLN can be grown to adequate numbers for ACT and harbor cells that are tumor specific [15]. In a study of metastatic colorectal cancer patients undergoing ACT with TDLN, there was an objective response in 6 of 32 patients (~19%), and median survival was 12.5 months compared to 5.8 months in historical controls [16].

Another source of T-cells is peripheral blood mononuclear cells (PBMC). This source is especially appealing because it does not rely on any surgical procedure (either tumor or lymph node excision) to obtain cells. In general, patients undergo leukapheresis to obtain a large sample of white blood cells from the peripheral blood. From this sample, there are several methods to generate tumor specific T-cells. One protocol, which has been used in clinical trials, is to separate leukapheresis samples into adherent and non-adherent cells [17]. The adherent cells are then used to generate autologous dendritic cells (DC). These DC are then pulsed with appropriate tumor antigens and incubated with non-adherent cells which are selected for CD8^+^ cells using magnetic beads. These stimulated T-cells are then clonally expanded in preparation for ACT. Generation of T-cells in this manner has demonstrated some efficacy in human trials of metastatic melanoma. In one study, five patients who underwent T-cell infusion also had immunohistochemical staining of their tumors pre- and post-infusion [18]. In three of these patients there was loss of the targeted antigen in recurrent or persistent melanoma. This indicates that T-cell infusion was effective against melanoma cells bearing the appropriate target, but unfortunately, melanoma cells without appropriate target were able to persist. Interestingly, a similar methodology was used to generate CD4^+^ T-cells specific for NY-ESO-1 antigen and treat a patient with metastatic melanoma [19]. In this report, the patient had a complete response even though only one antigen was targeted and only 50%–75% of the tumor expressed this antigen; leading to the thought that CD4^+^ T-cells may play a greater role in coordinating an immune response resulting in antigen or epitope spreading [20]. While this protocol had displayed efficacy in human studies, it was labor intensive. More recently, researchers have experimented with harvesting T-cells from peripheral blood and genetically engineering a TCR that will target a specific tumor antigen. Several trials have been performed showing the clinical effect of these cells [21,22]. Morgan et al. showed a response rate of 13%, while Johnson et al. showed a response rate of up to 30%. Most importantly, “on-target/off-tumor” autoimmunity for these genetically engineered cells was first reported.

Finally, genetic engineering may provide a near limitless source of T-cells from induced pluripotent stem cells (iPSC). While hematopoietic stems cells seem like an ideal source of stem cells for lymphocyte generation, they are difficult to grow in large numbers in culture and can have defects in patients with advanced age [23]. Pluripotent stem cells are a better source, but the gold standard cells are difficult to obtain as they come from embryonal tissues. Significant research has been performed into induced pluripotent stem cells, which are generated through gene transduction of somatic cells with four specific genes: Oct3/4, Sox2, c-Myc, and KLF4 [24]. These cells can then be used to generate T-cells, which have not undergone TCR gene rearrangement; therefore, they require further genetic modification to provide them with tumor-specificity [25]. Interestingly, early research into the use of induced pluripotency in this field also utilized mature antigen specific T-cells. These T-cells were transfected with the above mentioned genes to generate iPSC. These iPSC, however, maintained their T-cell receptor gene rearrangements and, therefore, their antigen specificity [26,27]. These experiments suggest a means to circumvent T-cell exhaustion as well as provide a method for the generation for near-limitless naïve/memory T-cells. Advancements in the generation of tumor-specific T-cells using iPSC are evolving, but currently there is little data regarding their clinical efficacy or safety.

## 3. Ex Vivo Conditions

ACT remains a treatment option at a limited number of specialized centers across the country. There are likely several reasons for this, however, one is that most ACT protocols require significant ex vivo processing of T-cells prior to treating patients. These manipulations and ex vivo processing must occur in an FDA certified Current Good Manufacturing Practices (cGMP) facility. Operating these types of facilities requires a significant investment and commitment by institutions. In addition, each source of T-cells requires its researchers and clinicians to have a particular expertise to establish the necessary number and efficacy of the cells, as well as management of the patient during the process of ACT.

The conditions under which T-cells are cultured is probably the most significant and easily manipulated ex vivo processing that is performed. The importance of ex vivo culturing conditions lies in the fact that the final product may be selected to favor replicative capacity, effector function, or memory generation depending upon the length of culture and the cytokine milieu. In general, TIL, as a prototypical ACT, has a straightforward if not labor-intensive protocol for ex vivo culturing. As described by Dudley et al., the first step in the generation of TIL for ACT is to excise tumors from patients with cancer [28]. These tumors are then cut into 1–2 mm^3^ pieces and either placed directly into a 24 well plate or disaggregated into a single-cell suspension prior to plating. The cells are then cultured in a lymphocyte medium, which usually contains about 10% human serum and high-dose IL-2 (6000 IU/mL) for about 1–2 weeks until there is sufficient TIL outgrowth from tumor cells. Individual cultures are then tested for tumor reactivity through overnight cytokine release assays with either autologous tumor, HLA-matched cell lines, or HLA-matched APC pulsed with tumor antigens. Those TIL cultures that show tumor reactivity by increased secretion of IFN-γ are then expanded with anti-CD3 antibody, IL-2 and allogeneic feeder cells for approximately 14 days, at which time they are then harvested and prepared for infusion into the patient.

While this process has yielded some impressive results in select patients, it takes about four weeks to convert the excised tumor sample into a lymphocyte product ready for infusion. Also a large percentage of patients do not have enough starting TIL to generate sufficient numbers for adoptive transfer or produce a TIL culture that secretes INF-γ [29]. One study shows that up to 38% of patients who had traditional TIL cultures did not possess enough IFN-γ secretion to ultimately undergo treatment [30]. Interestingly, expression of costimulatory molecules, telomere length and, persistence of cells in vivo are thought to be the strongest predictors of clinical response [31,32]. To that end, Besser and colleagues proposed a protocol of “young-TIL”, in which individual cell cultures are not selected for rapid expansion based on INF-γ production [30]. Young-TIL are bulk cultured from excised tumors for 10–18 days, which greatly shortens the culture duration and preserves the replicative capacity of the cells following adoptive transfer. In addition, these cultures are not selected prior to infusion, which allows more patients to obtain this therapy.

The use of TDLN is similar to that of young-TIL, in that there is no selection process for the cell cultures. Cells are generally grown in bulk cultures either to a desired number or for a set culture period. The shorter culture period for both TDLN and young-TIL is appealing for several reasons. First, shorter culture duration will favor cells that have undergone fewer cell divisions and therefore have longer telomeres. The implication is that after ACT with these cells there will be higher replicative capacity in vivo and longer persistence of adoptively transferred cells. Secondly, patients with metastatic cancer will usually need more timely treatment. They are at high risk for clinical deterioration and disease progression. Therefore, shorter culture duration may be able to get these patients to treatment in 2 weeks versus 4–6 weeks, which may be of added benefit.

While culture duration is an important characteristic in producing T-cells for ACT, there are other conditions worth considering. Basic knowledge of T-cell activation has grown immensely. Appreciation for the function of the TCR and the mechanism of activation of T-cells was blossoming in the 1980s [33]. These original concepts have grown into a more nuanced understanding of the TCR role in T-cell activation against an antigen or tolerance to the same antigen [34]. T-cell activation against its cognate antigen is currently viewed as a complex process involving 3 signals [35]. In general, signal 1 is the TCR binding to its cognate antigen in the context of a major histocompatibility complex (MHC) molecule. Signal 2 represents co-stimulatory molecules that function in parallel to Signal 1 to enhance (e.g., CD28) the activation of T-cells. Signal 2 also encompasses inhibitory signals (e.g., CTLA-1 and PD-1) that generate tolerance or anergy to the T-cell’s cognate antigen. Signal 3 is the cytokine milieu that the T-cell is exposed to during activation that can generate a graded response to its antigen. Ineffective immune responses to tumor cells can occur at any or all of these signaling steps to generate T-cell tolerance. The purpose of ex vivo culturing of T-cells for ACT is to generate an increased number of cells but also to remove the T-cells from the tumors immunosuppressive effects and have the ability to manipulate signals to the T-cells to generate the most effective anti-tumor responses. Therefore, manipulating these signals to maximize the benefit of ACT is an area of growing research.

Most ex vivo ACT protocols have a method of “activation” of T-cells. These methods typically start by recapitulating signal 1 either directly or indirectly. This is accomplished in vivo by T-cells binding to their cognate antigen presented by tumor cells or APC in the context of MHC I or MHC II molecules. This can be performed ex vivo as well. Utilizing autologous tumor, tumor cells lines or known antigenic peptides, T-cells and APCs can be cultured together to activate T-cells for ACT [36]. These T-cells and APCs have to be human leukocyte antigen (HLA) matched to effectuate T-cell activation. While this does provide for stimulation of only tumor-specific T-cells, it is a laborious protocol that requires significant time for culture. One of the most common mechanisms to mimic signal 1 and activate T-cells is to use an activating antibody to CD3. This is the initial step of the Rapid Expansion Protocol that was made popular by the NCI [28]. Using activating anti-CD3 antibodies is relatively easy, cost effective, and does not require additional cell culturing. This method activates all T-cells regardless of their antigen-specificity. Therefore, all T-cells with tumor-specificity will be activated without having to identify their specific antigen. However, T-cells that are not specific to tumor cells will be activated as well. The clinical relevance of activating non-tumor specific T-cells is debatable as patients who have received non-specifically activated TDLN have demonstrated minimal toxicity [16].

Manipulation of signal 2 has recently gained more interest, particularly with usage of checkpoint inhibitors (e.g., anti-CTLA-4 and anti-PD1 antibodies) in clinical practice. These antibodies block inhibitory signals to T-cells to abrogate anergy or tolerance. By performing ex vivo culturing of T-cells, inhibitory signal 2 effects are absent. However, studies have focused on the use of positive co-stimulatory signals ex vivo to generate more effective T-cells for ACT. Utilizing magnetic beads coated in both anti-CD3 and anti-CD28 antibodies to activate T-cells, Ito and colleagues demonstrated improved efficacy of ACT as compared to traditional plate-bound anti-CD3 antibody alone in a murine sarcoma pulmonary metastasis model [37]. Interestingly, they also showed that cells activated with the anti-CD3/CD28 beads had higher production of INF-γ and IL-2 than anti-CD3 alone. This may be due to the finding that anti-CD3/CD28 bead-activated cultures had higher percentages of CD4^+^ T-cells. Other studies have also focused on CD4^+^ T-cells as effectors of cancer immunotherapy, as lymph nodes harbor a mixed population of CD4^+^ and CD8^+^ T-cells. TIL classically utilizes only CD8^+^ T-cells as these cells infiltrate into the tumor in higher numbers and are the established effectors of direct tumor killing. Several reports have shown that CD4^+^ T-cells have efficacy in ACT either through supporting the direct cytotoxic function of CD8^+^ T-cells or via indirect CD4^+^ dependent mechanisms of tumor cell killing [38,39].

Signal 3 interventions are being investigated to determine their impact on the efficacy of T-cells for ACT. The cytokine milieu can be altered to affect the phenotype or the growth potential of T-cells. Although manipulation of the cytokine milieu has been of more recent interest in the field of ACT, this therapeutic modality would not have been possible without the discovery of the original T-cell growth factor, IL-2, in 1976 [4]. Use of IL-2 is a routine part of all T-cell cultures, however, different protocols use varying concentrations. While traditionally “high-dose” IL-2 (at concentrations up to 6000 IU/mL) was used in T-cell cultures [28], some authors have shown significant and consistent culture growth with concentrations as low as 100 IU/mL [15]. The implications of using high versus low dose IL-2 in T-cell culture are not totally understood. Most T-cell cultures using high dose IL-2 are CD8^+^ T-cell TIL cultures which may have a higher requirement for IL-2. On the other hand, TDLN cultures that have a mixed population of CD4^+^ and CD8^+^ T-cells may produce enough of their own IL-2 that exogenous doses can be reduced.

As more cytokines have been discovered and their functions elucidated, researchers have begun to study how they can influence the development of optimal cells for ACT. Several studies have shown that terminally differentiated effector T-cells are not the optimal cells to use in ACT [40,41]. Both CD4^+^ and CD8^+^ T-cells can be sub-divided based are their level of differentiation from a naïve T-cell to an effector T-cell [42]. Additionally, differentiation into subsets of central memory T-cells (T_CM_) and effector memory T-cells (T_EM_) is possible. Gattinoni et al. also reported on a subset of T-cells termed stem cell memory T-cells (T_SCM_) which appear to have features of both naïve and memory T-cells [43]. These memory cells can become effector cells in vivo but will also maintain a circulating reserve of cells that persist in the circulation. Memory subsets may allow for persistence which is a key characteristic associated with a maintained response to ACT [4]. In contrast, a major concern of high-dose IL-2 is the generation of terminally differentiated T-cells that have limited replication potential following adoptive transfer. Therefore, signal 3 manipulation ex vivo may have a profound influence upon the lymphocyte product and subsequent clinical outcome.

With further understanding of in vivo T-cell homeostasis, IL-7 has emerged as an important cytokine in the persistence of naïve and memory T-cells [44]. Examining the role of IL-7 in T-cell culture has shown that the addition of IL-7 will keep T-cell cultures viable beyond 5–6 weeks [45]. Importantly, these cells also maintain their activity against in vivo established tumors even with greater than 12 months of continuous culturing. As most ACT protocols do not culture cells for more than 6 weeks, the clinical implication of prolonging cultures is unknown. However, as researchers begin to study the role of repeated infusions of T-cells, IL-7 may offer the potential for long term continuous cultures for repeated adoptive transfers. IL-15 has also been shown to have a role in proliferation and maintenance of several cell lines, including T-cells [46]. Klebanoff utilized IL-15 to generate CD8^+^ T_CM_ cells and compared these with IL-2 generated CD8^+^ T_EM_ cells [47]. This study showed that IL-15 leads to differential gene expression that increased the T-cells’ ability to traffic to secondary lymphoid tissues, a feature of T_CM_. These cells also were more effective in treating established B16 melanoma tumors in mice and resulted in significantly prolonged survival. Additionally, IL-21 has shown a role in directing the differentiation of T-cells used in ACT. IL-21 enhances effector functions of T-cells but also plays a role in the development of memory T-cells [48]. Using IL-21 during the priming of CD8^+^ T-cells suppresses the differentiation of T-cells effector functions, as shown by decreased expression of Eomes and increased expression of Tcf7 [49]. IL-21 resulted in the best response to established B16 melanoma tumors as compared to IL-2 and IL-15. Interestingly, in this study, IL-15 resulted in increased expression of effector function genes similar to IL-2. The reason for the discrepancy between this study and Klebanoff’s work is unknown, but may be related to differences in the starting cells. In studies using IL-15, bulk CD8^+^ splenocytes were used, while in the studies of IL-21 naïve CD8^+^ splenocytes were selected prior to culturing. Some authors have suggested the use of naïve T-cells for both increased efficacy of ACT as well as better control of differentiation in vitro [50].

In addition to manipulating cytokines to effect T-cell differentiation, cytokines play an important role in the functional subset of T-cells. Effector T-cell responses have been divided into subtypes, Th1 and Th2. Although highly complex in terms of lymphokine secretion, Th1 responses generally favor INF-γ with Th2 responses favoring IL-4 [51]. While these subsets were initially described in CD4^+^ T-cells, CD8^+^ T-cells also follow a similar functional pattern, Tc1 and Tc2 [52]. Aruga demonstrated that anti-tumor responses are generated by T-cells expressing a Th1/Tc1, or type 1, functional pattern but not by T-cells expressing a Th2/Tc2, or type 2, pattern [53]. Cytokines that promote a type 1 immune response are typically preferable as this response increases the anti-tumor activity of cells in ACT. IL-12 and IL-18 have emerged as type 1 promoting cytokines. When used in combination, IL-12 and IL-18, have been shown to be synergistic in generating a type 1 response in cultures of TDLN [54]. When these were used for ACT in a sarcoma pulmonary metastases model they showed significant reduction in disease burden. Interestingly, maximal response required treatment with both CD4^+^ and CD8^+^ T-cells. In addition to Th1 and Th2 pathways, Th17 and Treg pathways have been described [55] which suggests that influences and factors present during T-cell activation and differentiation may generate a myriad of cell phenotypes each with unique characteristics.

Ex vivo activation of T-cells as part of ACT protocols was initially thought to remove cells from the immunosuppressive effects of tumor-bearing hosts. However, it also removes cells from physiologic mechanisms to activate T-cells via signal 3. The activation of T-cells via a CD3 antibody (even with addition of CD28 activation) is a generalized signal that does not provide a “program” to the T-cell. By providing cytokine stimulation at the time of ex vivo activation a more nuanced response from the T-cells can be generated. Various studies have looked at the ability to generate memory cells or an anti-tumor T-cell phenotype, however, no definitive data has elucidated the ideal cytokine signal(s). The optimal recapitulation of signals 1, 2, and 3 to generate anti-tumor lymphocytes is complicated not only by the multitude of combinations of factors but also by the timing and dosing of such factors, and to date, these parameters remain unknown.

## 4. Genetic Engineering

Due to the encouraging results of studies of ACT in melanoma, similar trials in various other malignancies have been attempted. Unfortunately, these trials have yielded less encouraging results. One of the reasons is that while it is feasible to harvest TIL from melanoma, this is more difficult in other tumors that may require a more extensive surgical procedure. Also, TDLN from intra-abdominal tumor sites (e.g., colorectal cancer) may be difficult to identify and harvest. These factors have led researcher to pursue other sources of tumor-specific T-cells. While researchers have used PBMC in early ACT protocols, their greatest use has been as a substrate for the generation of genetically engineered T-cells.

In 1991, van der Bruggen was able to identify the antigen in a patient with melanoma that T-cells were specific to and subsequently clone the antigen gene—MAGE1 [56]. This was a major breakthrough in ACT therapy and subsequently, many other antigen genes have been cloned from the tumor-specific TIL found in tumors. These tumor-associated antigens (TAAs) can either be related to tissue differentiation, tumor-testis antigens, or normal proteins that are highly overexpressed [57]. With the ability to clone these genes, researchers could rely on classic molecular biological techniques to generate tumor-specific T-cells. Using PBMC, or more specifically peripheral blood lymphocytes (PBL), a large number of lymphocytes can be obtained from almost any patient. With an abundant source of cells, most of the technical considerations focus on the ability to introduce a foreign gene into the T-cell. Van der Bruggen used electroporation to introduce the exogenous gene into lymphocytes; however, others have used viral vectors [58] to increase the efficiency of transfection and generate a modified TCR. A classical TCR is composed of an α and β chain that form a heterodimer. Each of these subunits contributes to the antigen specificity of the TCR. In these protocols, the native TCR is not altered, and therefore remains on the surface of the T-cell. The gene modified TCR is functionally the same as the native TCR in that it requires its antigen to be presented in the context of an HLA-matched MHC molecule. The T-cell is also unaltered in its response to co-stimulatory or inhibitory molecules.

In the first clinical trial of genetically engineered T-cells, Morgan treated 15 patients with T-cells harvested from peripheral blood and transfected with the genes encoding the α and β chains of a MART-1 TCR [21]. Two patients in this study demonstrated an objective response to treatment and were noted to have the genetically engineered T-cells persist in peripheral blood for over 1 year. Importantly, none of these patients experienced significant adverse effects related to T-cell therapy. While these results were encouraging, responses were less impressive as compared to those described for other ACT therapies. In a follow-up study using a TCR with higher avidity, Johnson demonstrated a 30% objective response rate for a human TCR and 19% objective response rate for mouse TCR [22]. However, in this study autoimmune anterior uveitis and ototoxicity were observed in approximately 47% of patients and did not correlate with an anti-tumor response. This is in contrast with TIL therapy, in which fewer than 10% of patients experience similar toxicities.

There are several drawbacks to the use of gene-modified TCR T-cells. One of the most significant is that many cancers down-regulate their MHC molecules as a mechanism for immune escape [59]. Since gene-modified TCR T-cells are MHC restricted and target only a single antigen, this limits their therapeutic efficacy. Additionally, these T-cells are also subject to the inhibitory signals or lack of co-stimulatory signals that are expressed on the surface of tumors, which can alter their in vivo activation. Finally, exogenous α and β chains can mispair with endogenous genes and result in auto-reactive TCRs [60].

To address these limitations of gene-modified TCR T-cells, chimeric antigen receptor (CAR) T-cells have been increasingly utilized. CAR T-cells are distinct from other forms of ACT in that they do not use the TCR machinery for antigen-specificity. The CAR is generated by using a variable fragment of a monoclonal antibody to target a specific antigen. This extracellular variable region is then linked to intracellular domains that contain activation domains from the CD3 gene via a transmembrane anchoring region [61]. Unlike TCRs which recognize peptide sequences, the variable region of CAR functions as an antibody, and therefore can recognize protein, lipid, or carbohydrate motifs. Second- and third-generation CAR incorporated intra-cellular costimulatory domains to enhance the activation and persistence of T-cells [62]. Studies have shown that different costimulatory domains can have a significant impact on CAR T-cell efficacy. Kawalekar et al. demonstrated that utilizing the 4-1BB costimulatory domain as opposed to the CD28 domain results in T-cells with increased memory cell functions and prolonged survival—both of which have been shown to be more effective in ACT [63]. CAR T-cells have shown promise especially for the treatment of hematologic malignancies [64,65].

Both gene-modified TCR T-cells and CAR T-cells can have “on-target/off-tumor” toxicities due to recognition of antigen on normal tissue. Unfortunately, CAR T-cells also have adverse events that can be severe or life-threatening. Cytokine release syndrome is a unique complication of CAR T-cells. It occurs with the significant release of cytokines that results from synchronous activation of T-cells. These cytokines (TNF-α, INF-γ, and IL-2) can result in hypotension, hypoxia and neurologic symptoms [66]. Also, some CAR T-cells are generated using murine antibodies in the variable region. These T-cells are known to lose expression of the CAR. Therefore, some researchers have examined the role of repeated infusions. In one study, a patient undergoing CAR T-cell therapy utilizing a murine variable region developed a life-threatening anaphylactic reaction [62]. Continued efforts in the field of CAR T-cells ACT are focused on both increased efficacy and safety.

Genetic engineering has been performed to insert genes to alter the function of T-cells with conventional TCRs. Zhang and colleagues utilized TIL grown under standard culture conditions, but inserted an IL-12 gene under the control of the nuclear factor of activated T-cell (NFAT) inducible promotor [67]. When used for ACT, these cells were found to be efficacious without the use of exogenous IL-2. 33% of patient demonstrated an objective response with a clear dose-response relationship. However, this study also showed significant toxicities of IL-12 producing cells. Many patients developed grade 3 liver toxicity and some patients had such elevated levels of IL-12 in their serum that they required monitoring in an ICU setting.

Another approach to genetically engineered T-cells is to extend their longevity. As discussed previously, the persistence of T-cells is a significant factor in determining clinical response. One of the limiting steps to any cell’s proliferative capacity is the length of its telomeres. Several authors have attempted to address this by using T-cells transduced with the human telomerase reverse transcriptase (hTERT) gene. These T-cell clones have been shown to have stable telomere length through 170 population doublings while maintaining their cytotoxic properties [68]. These hTERT-transduced T-cells maintain the expression of hTERT regardless of activation status and with some suggesting that these cells can be considered immortalized [69]. While the clinical benefit of prolonged persistence of T-cells is obvious, the implications of an immortalized cell for use in ACT give rise to much more serious concerns. Uncontrolled growth and the possibility of malignant transformation of ACT cells preclude the use of hTERT-transduced cells in clinical practice. To address this issue, “suicide genes” have been introduced into hTERT-transduced T-cells. Two systems are the herpes simplex virus-thymidine kinase (HSV-Tk) gene and the *E. coli*-nitroreductase (*E. coli*-Ntr) gene [70]. These genes work by converting a pro-drug (ganciclovir for HSV-Tk and metronidazole for *E. coli*-Ntr) into a toxic metabolite the precipitates cell death. While these may prove to be effective measures to prevent uncontrolled growth by hTERT-transduced T-cells, multiple gene transductions add to the complexity of generating these cells for clinical use.

## 5. Conclusions

Although ACT is a treatment modality that has been used for decades, successful research continues to highlight new aspects of this therapy. The sources of T-cells used to generate cultures for ACT have been significantly expanded. Each source of cells has distinct advantages and limitations, such that future ACT may rely on a patient- and cancer-centered approach as opposed to a one-size-fits-all method of ACT. While the source of T-cells has generated intense interest, the ex vivo culturing and manipulations of these cells have truly been revolutionized since the first cultures for ACT. Active research into altering all aspects of T-cell activation and signaling is being performed. These studies have generated significant findings that may be applicable to both ACT and other forms of immunotherapy. Gene therapy has also found its use in ACT. From inserting off-the-shelf TCRs or modifying the cytokine production of T-cells, genetic engineering of T-cells has proven to be a powerful tool. A recent example of this is the use of whole exome sequencing (WES) to truly tailor the T-cell and TCR to the patient’s cancer [71].

ACT is often used as an umbrella term as if it were a single therapy. However, there is a myriad of ACT methods based on cell source and ex vivo manipulations. Including the various forms of patient pre-conditioning and adjuvant treatments, ACT as a tool in the armamentarium of immunotherapy has continued promise.
